# Why and How the Indo-Mediterranean Diet May Be Superior to Other Diets: The Role of Antioxidants in the Diet

**DOI:** 10.3390/nu14040898

**Published:** 2022-02-21

**Authors:** Ram B. Singh, Jan Fedacko, Ghizal Fatima, Aminat Magomedova, Shaw Watanabe, Galal Elkilany

**Affiliations:** 1 Department of Medicine, Halberg Hospital and Research Institute, Moradabad 244001, India; 2 Centre of Clinical and Preclinical Research-MEDIPARK, Pavol Jozef Safarik University, 040-11 Kosice, Slovakia; 3 Department of Biotechnology, Era’s Lucknow Medical College and Hospital, Lucknow 226003, India; ghizalfatima8@gmail.com; 4 Department of Population, The Faculty of Economics, Lomosonov Moscow State University, 119991 Moscow, Russia; amgerma@gmail.com; 5 Department of Life Science Association, Tokyo 160-0005, Japan; watashaw@medicalrice.com; 6 Department of Cardiology, Gulf Medical University, Ajman 4184, United Arab Emirates; galal.elkilany@gmail.com

**Keywords:** Mediterranean diet, DASH diet, vegetables, fruits, hypertension, diabetes

## Abstract

The Seven Countries Study showed that traditional Japanese and Mediterranean diets are protective against cardiovascular diseases (CVDs). The Japanese diet is considered the healthiest because it provides Japanese populations with the highest longevity and health. DASH and Mediterranean-style diets are also considered healthy diets, although the Indo-Mediterranean-style diet may provide better protective effects among patients with CVDs compared to other diets. The concept of the Indo-Mediterranean type of diet was developed after examining its role in the prevention of CVDs in India, the value of which was confirmed by a landmark study from France: the Lyon Heart Study. These workers found that consuming an alpha-linolenic acid-rich Mediterranean-style diet can cause a significant decline in CVDs and all-cause mortality. Later in 2018, the PREDIMED study from Spain also reported that a modified Mediterranean-style diet can cause a significant decline in CVDs, type 2 diabetes mellitus (T2DM), and cancer. The Indo-Mediterranean diet may be superior to DASH and Mediterranean diets because it contains millets, porridge, and beans, as well as spices such as turmeric, cumin, fenugreek, and coriander, which may have better anti-inflammatory and cardioprotective effects. These foods are rich sources of nutrients, flavonoids, calcium, and iron, as well as proteins, which are useful in the prevention of under- and overnutrition and related diseases. It is known that DASH and Mediterranean-style diets have a similar influence on CVDs. However, the Indo-Mediterranean-style diet may be as good as the Japanese diet due to improved food diversity and the high content of antioxidants.

## 1. Introduction

The Seven Countries Study was the first to observe that the people of Japan and Mediterranean countries have a low risk of cardiovascular diseases (CVDs), which may be due to the cardioprotective effects of their diets [1,2,3]. The UNO in 1998 certified that the Japanese diet could be a crucial factor for their longevity and healthy life expectancy. In Mediterranean countries such as Greece, Southern Italy, and Spain, people consume a traditional Mediterranean diet, which is protective against CVDs, diabetes, and cancer [4]. In addition to these diets, the DASH diet has been developed and found to be protective against CVDs [5,6]. It seems that there are both similarities and discrepancies in the consumption of foods and nutrient intake, in particular antioxidant and w-3 fatty acid intake, which could be a determinant of the effectiveness of these diets [3,4]. A traditional Japanese diet shares several similarities with a traditional Mediterranean diet, as both are based on a foundation of plant foods such as rice, vegetables, grains, legumes, and fruit. Fish is consumed regularly, more in Japan than in Mediterranean countries, while red meat is consumed more sparingly. The majority of fat consumed in Japan is rice bran oil, whereas in Mediterranean countries, it is olive oil and from unsaturated sources [3,4]. It has been found that all special types of diets may safeguard against the risk of CVDs and type 2 diabetes mellitus (T2DM) [4,5,6,7,8]. The Japanese diet is naturally rich in fish, seaweed, green tea, soy, fruits, and vegetables and low in added sugar, fat, and other animal protein. However, the Indo-Mediterranean diet is higher in whole grains, spices, and mustard oil, in addition to fruits and vegetables and fish to nonvegetarians. The Indo-Mediterranean diet has been found to be protective against CVDs [7,8,9,10]. It is proposed that whole grain content, in particular antioxidants in various diets, may explain, at least in part, the differences in the cardioprotective potential of these diets. This article aims to highlight the differences in the efficacy of these diets.

## 2. Comparison of Various Types of Healthy Diets

Epidemiological studies, including cross-sectional surveys, long-term prospective observational studies, and short-term trials of intermediate outcomes, have provided supporting evidence that western-type diets have adverse effects, whereas diets rich in nutrients with a modest amount of fish are protective against CVDs, T2DM, and cancer [1,11,12,13,14,15,16]. All high-quality diets are characterized by unrefined, unprocessed, or minimally processed foods with a high glycemic index. The major differences in various types of diets are determined by several food items such as fruits, vegetables, nuts, fish, and vegetable oils, which are beneficial and rich in nutrients. However, western types of foods, consisting of refined and fast foods, syrups, red meat, processed meat, salt, sugar and trans fat, have been found to have potential causal relationships between specific dietary factors and NCDs (coronary artery disease, diabetes, and colorectal cancer) [1,16,17,18]. Japanese and Mediterranean diets, as well as the DASH diet, are known to be healthy [4,5,6,18]. The populations in Mediterranean countries have a lower risk of CVDs, T2DM, and cancer, which is similar to Japanese populations, who are known for longevity, as well as a healthy life expectancy [3,17,18]. The Mediterranean diet is characterized by greater consumption of whole grains, (wheat), fruits, and vegetables, along with olive oil and fish [4,17,18]. The DASH diet shares some similarities with the Mediterranean diet because it also contains more vegetables, nuts, whole grains, and fish, with less refined foods and less red and preserved meat and poultry, without any emphasis on olive oil [5,6]. Japanese populations also consume large amounts of whole grains (rice), vegetables, fruit, and fish, but there is a much lower intake of energy from oils and fats [3,17,18]. The traditional Mediterranean diet includes high amounts of total fats (80–100 g/day) with 40% of energy from polyunsaturated fatty acids (PUFAs) in males, with high proportions of monounsaturated fatty acids (MUFAs), largely from olive oil and nuts [4,17,18]. However, the typical Japanese diet has been characterized by lower consumption of total fats (15–20% energy kcal/day), including saturated fatty acids, MUFAs, and PUFAs, particularly *n*-6 PUFAs [3,17,18]. Interestingly, over the last 2–3 decades, there has been a change from 20% energy from fats to 30%, whereas the ratio of *n*-6 PUFAs/*n*-3 PUFAs has shifted from 2–3 to 4–5 [1,2,3,17,18]. These alterations in Japanese dietary patterns may enhance the risk of CVDs and T2DM in Japan. There is an unmet need to determine the effects of changes in the concentrations of fatty acids and western-type foods and nutrients in the Japanese diet on the health and risk of CVDs [11,12]. The differences in various types of high-quality diets are shown in Table 1.

The Indo-Mediterranean-style diet differs because it contains more whole grains, in particular millets, porridge, and green beans, and a variety of healthy spices such as turmeric, coriander, cardamom, cinnamon, cumin, black pepper, cloves, etc. This diet can prevent the double burden of diseases due to undernutrition and overnutrition.

## 3. Antioxidants in Foods of Various Diets

Any agent with the ability to either quench free radicals or prevent the generation of pro-oxidant molecules, such as reactive oxygen species (ROS), can be considered an antioxidant [19]. In humans, the main source for the production of ROS is the mitochondria, which can generate hydrogen peroxide, singlet oxygen (1O2), or superoxide radicals (O2•−) [15,19,20]. However, in plants, it is the chloroplast that is the crucial organelle to produce ROS. Each cell in tissues and organs has its own independent antioxidant components to quench the deleterious effects of their internal ROS levels and production. Catalase, superoxide dismutase (SOD), glutathione peroxidase (GPS), and ceruloplasmin are the most powerful natural antioxidants produced in the body for protection against ROS [19]. Decreased intake of minerals, iron, zinc, selenium, and copper may worsen the deficiency of these antioxidants because these minerals are required for their synthesis [20]. Apple, grapes, watermelon, orange, guava, and green leafy vegetables are rich sources of antioxidant nutrients such as vitamins A, C, and E, as well as the minerals copper, zinc selenium and beta-carotene, lutein, and lycopene [11,12,15]. Whole grains are rich sources of magnesium, calcium, iron, folic and minerals (millets), and all beans provide adequate proteins. Fruits, vegetables, nuts, and whole grains are all rich in flavonoids [2,11,12,19,20].

### 3.1. Flavonoids in Foods and Diets

Flavonoids, flavones, catechins, polyphenols, anthocyanins, lignans, and phytoestrogens are all types of antioxidants and phytonutrients, and they are all found in plant-based foods [19,20]. There are more than 4000 known flavonoids, which are just one class of antioxidants. Diets rich in berries such as blackberries, cranberries, strawberries, or blueberries are the richest sources of antioxidant nutrients, in particular anthocyanins [15]. Anthocyanins are also from the flavonoids group, which are pigments widely distributed in fruits and vegetables [19]. Anthocyanins are responsible for red, blue, purple, and yellow colors in fruits, flowers, and vegetables and protect plant cells from the stresses of the environment such as high sunlight and pollutants [20]. In a recent study on the flavonoid content of foods, lingonberries, and blueberries, they were found to contain a greater amount of flavonoids (1100 mg/100 g dry weight) than raspberries and strawberries (500 mg/100 g dry weight). Anthocyanins were the dominant flavonoids in all berries [21]. This analysis reported compounds contributing varying amounts of the total flavonoid content in various types of strawberries (18%), lingonberries (29%), raspberries (^1%), and blueberries (67%). In eastern and western societies, tea, coffee, cocoa, and wine are the primary dietary sources of flavonoids. In addition, leafy vegetables, berries, apples, cherries, soybeans, onions, and citrus fruits are considered important sources of dietary flavonoids [15,20,21,22,23,24,25,26,27,28,29,30].

The USDA was the first to release a database on the flavonoid content of foods in 2003, which was updated using new data on 20 different flavonoids from a nationwide sampling of 59 fruits, nuts, and vegetables and 102 scientific papers [22]. The new database contains data on flavonoids for 395 food items and data from the National Health and Nutrition Examination Survey to ascertain the intake of five classes of flavonoids: anthocyanidins, flavanones, flavonols, flavones, and flavan-3-ols on a population basis (www.ars.usda.gov/nutrientdata). Interestingly, black tea provided the largest amount of flavonols to the diet (32%), followed by onions (25%), and parsley was the largest contributor of flavones. Dried parsley leaves contain a large amount of 13.53 g/100 g, though rarely is 100 g consumed at one time, and a teaspoon weighs 0.5 g. Among fruits, oranges (53%) and grapefruit juice (16%) contributed significant amounts of flavanones. Brewed tea provides the largest quantities of flavan-3-ols to the diet. Blueberries contributed the largest amount of anthocyanidins (31%), followed by bananas (21%) and strawberries (14%). Although bananas contain a lower amount of anthocyanidins than any of the berries, the intake of bananas is much higher than that of individual berries. This database provides worldwide researchers with new values on the flavonoid content of many more foods in order to better ascertain the impact of flavonoid consumption in various diseases [22]. Daily per capita intake of flavonoids in the United States using these data was: anthocyanidins, 5 mg; flavanones, 4 mg; flavones, 1 mg; flavonols, 10 mg; flavan-3-ols, 112 mg; total intake 132 mg/day. This is too low compared to the estimated flavonoid intake in European (250–900 mg/day), Asian (200–650 mg/day), and Middle Eastern countries (1650 mg/day) [15]. The higher intake of flavonoids in these countries is mainly because of black tea [22,23,24]. In the Indo-Mediterranean diet, the flavonoid intake was 1800 mg/day [7,9,10]. Despite the high intake of fruits, vegetables, and red wine in Mediterranean countries, the intake of total flavonoids in these countries (250–400 mg/day) is lower than in other European countries (350–900 mg/day), which is due to the much higher consumption of tea in non-Mediterranean countries [24]. In Japan, the mean intake of total flavonoids flavan-3-ols, isoflavones, flavonols, flavanones, and flavones was approximately 1500 mg/day, mainly from green tea, soya foods, onion, leafy vegetables, and fruits [25]. Thus, the total flavonoids in the Japanese diet are comparable with flavonoid contents in the Indo-Mediterranean diet (1500 versus 1800 mg/day, respectively), which makes these diets potential sources of flavonoid consumption for cardiometabolic protection [7,25].

### 3.2. Flavonoid Intake and Risk of Cardiovascular Diseases

Flavonoids are polyphenolic plant metabolites that have biologically relevant protective effects in a number of cardiometabolic disorders (CMDs) [26]. Some epidemiological studies have underscored a negative association between dietary flavonoid consumption and the propensity to develop CMDs, indicating that the contribution of the gut microbiota may be crucial for metabolizing dietary intake, as it is related to CVDs [26]. The Seven Countries Study reported that the consumption of flavonoids at baseline in the year 1960 was estimated by flavonoid content analysis of equivalent food composites of the diets in the 16 cohorts [2]. After follow up of 25 years, mortality from CAD, cancer in various organs, and all causes in the 16 cohorts revealed that average consumption of antioxidant flavonoids was inversely associated with CAD mortality. The study also explained about 25% of the variance in the rates of CAD in all the 16 cohorts. The consumption of saturated fat (73%; *p* = 0.0001), flavonoids (8%, *p* = 0.01), and percentage of smokers per cohort (9%; *p* = 0.03) together explained the independent consumption of alcohol and antioxidant vitamins, 90% of the variance in the rates of CAD. Interestingly, consumption of flavonoids was not independently associated with mortality from other causes. It is clear from this study that average flavonoid consumption may partly contribute to differences in mortality due to CAD across populations, but it did not seem to be an important determinant of cancer mortality in this study [2]. In a systematic review, a total of 39 prospective cohort studies were included, comprising 1,501,645 subjects and a total of 33,637 cases of CVD, 23,664 of CAD, and 11,860 of stroke [27]. Increased intake of total dietary flavonoids was linearly associated with a lower risk of CVD. Among the main classes of flavonoids, increasing intake of anthocyanins and flavan-3-ols is inversely associated with risk of CVD and flavonols and flavones with CAD. Only increasing flavanones showed a linear inverse association with stroke risk. Catechins showed a favorable effect on all outcomes of CVDs [27]. The intake of quercetin and kaempferol was also linearly associated with a lower risk of CAD and CVD, respectively. However, higher intake of all the aforementioned compounds was associated, to varying degrees, with a lower risk of CVD when comparing extreme categories of consumption. It seems that a flavonoid-rich diet may have potential cardiovascular benefits [27].

In another systematic review, at least 27 prospective cohorts (in 44 publications) evaluated the association between estimated habitual flavonoid intake and CVD risk [28]. The totality of evidence suggested that a long-term intake of flavonoid-rich foods may be associated with a lower risk of fatal and non-fatal CAD, CVD, and total CVD outcomes. The types of flavonoid subclasses more often included diets rich in anthocyanins, flavan-3-ols, and flavonols in lowering the risk of CVDs [28]. In the Nurses’ Health Study (1990–2018), including 60,582 women, and the Health Professional follow-up study (1990–2018), including 31,801 men, the follow-up period was 28 years [29]. After follow up, 36,856 deaths occurred. The multivariable-adjusted pooled HR for all-cause mortality among participants who had the highest consumption of olive oil (>0.5 tablespoons/day or >7 g/day) was 0.81 (95% CI: 0.78–0.84) compared with those who never or rarely consumed olive oil. The consumption of a greater amount of olive oil was associated with a 19% lower risk of CVDs mortality (HR: 0.81; 95% CI: 0.75–0.87), 17% lower risk of cancer mortality (HR: 0.83; 95% CI: 0.78–0.89), 29% lower risk of neurodegenerative disease mortality (HR: 0.71; 95% CI: 0.64–0.78), and 18% lower risk of respiratory disease mortality (HR: 0.82; 95% CI: 0.72–0.93). Replacement of 10 g/day of margarine, butter, mayonnaise, and dairy fat with the equivalent amount of olive oil was associated with an 8–34% lower risk of total and cause-specific mortality [29]. Interestingly, no significant associations were observed when olive oil was compared with other vegetable oils combined, indicating that it is the flavonoid content of olive oil that provided the benefits.

In a previous systematic review, total polyphenol consumption for the total population was assessed to be approximately 900 mg/day, which varied as per variations in the target groups of populations [23]. The main sources of polyphenols in the foods were vegetables, fruits, tea, coffee, and red wine. The intake of total flavonoids with specific subclasses, but without total polyphenols, was associated with a low risk of diabetes, CVDs, and all-cause mortality. Despite several variations in the data available, it seems that the concept of the beneficial effect of a dietary pattern, rich in polyphenol needs further studies in order to define specific therapy [23]. Interestingly, this review reported a higher intake of flavonoids compared to other studies [15,19,20,21,22,23,24,26,27,28,29]. Twelve studies found an inverse association between polyphenol intake and CV events [23]. A significant decline in CV risk was observed at the highest quartile of total polyphenol consumption in a few studies (1170 mg/day for Spain and 2632 mg/day for Poland) [23,30,31]. However, more research conducted in Spain and Iran (1248 mg/day and 2459 mg/day, respectively) found no benefits, which may be due to no substantial differences in the interindividual intake of flavonoids [32,33]. Other studies (*n* = 10) evaluating the association with intake of total polyphenol showed that only three of them had a significant inverse relation with CV events [32,34,35], with intake in the range of 115 to 944 mg/day. In the USA, an inverse association was observed for both CV and T2D, with the highest quartile of total flavonoids (585 mg/day) [34]. In Poland, total polyphenol intake of 2632 mg/day showed an inverse association with risk of T2DM [36]. In a population-based study in Brazil, an inverse association between polyphenol intake and hypertension was reported [30]. Interestingly, both diets, the Mediterranean and Japanese, are considered healthy, hence the people of Mediterranean countries have a low risk of CVDs and diabetes, while those of Japan are known to have greater longevity.

However, there are both similarities and discrepancies in intake of foods and beverages between the two diets. The Mediterranean diet is rich in cereals (wheat), fruits, vegetables, nuts, fish, and olive oil [4]. The Japanese diet consists of a large quantity of grains (rice), fish, raw fish, vegetables, and fruit and lower consumption of energy and total fats [1,2,3].

Mediterranean populations consume high amounts of total fats, mainly olive oil (approximately 100 g/day in males and 80 g in females) and polyunsaturated fatty acids [4]. In contrast, the traditional Japanese diet contains a lower amount of fat, However, the recent past has seen a change from 20% energy from fats to 30%, whereas the ratio of *n*-6 PUFAs/*n*-3 PUFAs has increased from 2–3 to 4–5, with no significant increase in CVDs [3]. It seems that the healthiest diet should have all the ten qualities of the diets and it should also involve mastication [8] (Table 2).

## 4. Determinants of Superiority of the Indo-Mediterranean Diet

The major constituents responsible for the superiority of the Indo-Mediterranean diet are more whole grains, such as millets, porridge, beans, brown rice, spices (coriander, turmeric, fenugreek, cumin, and cinnamon), peppers, onion, garlic, curd, and buttermilk, and lack of animal foods, except fish. Compared to other diets, it has all the ten special qualities [8,37,38,39,40]. The characteristics of these diets are not so much in the Mediterranean and DASH diets, as well as in the Japanese diet, due to the presence, although low, of red meat, preserved meat, refined foods, and egg [3,4,5,6]. The superiority of the Indo-Mediterranean diet over other cardioprotective diets is also clear from randomized trials, which have found that treatment with such diets was associated with a significant decline in CVDs, including heart failure and arrhythmias, as well as all-cause mortality [7,8,9,10]. Adverse effects of animal foods and refined foods have also been reported more recently in cohort studies [41].

The Indo-Mediterranean diet has no animal foods (except fish), lower saturated fat and total fat, sugar, and salt, and high omega-3 fatty acids and flavonoids. Some of these foods are known to activate nitric oxide release, which may be protective against CVDs, as well as diabetes [1,2,3,6,7,8,9,10,11,12,13]. A meta-analysis of studies comprising three randomized, single-blind trials examined the effects of Indo-Mediterranean-style foods in the prevention of CVDs, including heart failure [7]. These studies compared the intervention and control groups for behavioral risk factors, food intake, fatty acid intake, and ratio of polyunsaturated fatty acid (PUFA)/flavonoid intake (*n* = 1446 vs. 1320). The findings showed a significant protective effect of Indo-Mediterranean-style foods on heart failure and arrhythmias, which were similar to the Mediterranean diet. The Indo-Mediterranean diet also included fish intake (100–150 g twice weekly) for nonvegetarians, which has been found to reduce all-cause mortality [13]. Fruits and vegetables are rich sources of potassium, magnesium, and fiber, whereas whole grains such as beans and millets are rich in fiber, protein, polyphenolics, flavonoids, magnesium, calcium, iron, and folic acid. These nutrients can also take care of the double burden of diseases [8,9,10]. It is clear that the Indo-Mediterranean diet is superior to these diets because it has no animal foods, except fish, and it has a low glycemic index and high food diversity compared to all other protective diets.

There is evidence that a diet rich in healthy, plant-based foods and with fewer animal source foods such as fish, other sea foods, and poultry (up to five servings of animal source foods per week) can confer both improved health and environmental benefits. These dietary patterns are commonly observed in all protective diets such as DASH and Mediterranean-style diets, as well as in the Japanese diet [4,5,6]. Some experts believe that the diet used in the Dietary Advice to Stop Hypertension (DASH) study may be superior to the conventional Mediterranean diet [4,5,6,12]. The DASH diet is rich in fruits, vegetables, and dairy products with low fat and also contains whole grains, fish, poultry, and nuts and limited saturated fat, red meat, sweets, and sugar-containing beverages [5,6]. Thus, this diet is composed of lower amounts of total and saturated fat and cholesterol while providing higher amounts of potassium, magnesium, calcium, fiber, and protein. Despite minor differences between DASH and Mediterranean diets, few researchers have found other protective effects of this diet such as the reduction of hyperglycemia and insulin resistance, indicating that this diet can also be useful for the prevention of CVDs [4,5,6,11,12]. The traditional Mediterranean-style diet places greater emphasis on a high intake of olive oil, nuts, fruits, vegetables, and grains; moderate consumption of fish and poultry; and lower consumption of red meat, sweets, dairy products, processed meat with moderate consumption of wine [4,17,18]. Randomized controlled trials have found that increased adherence to the Mediterranean-style diet resulted in a positive effect on CVD risk and mortality [4].

In a systematic review among adults and older adults aged 17–84 years from 28 developed countries, the findings suggested that dietary patterns, possibly rich in flavonoids, including higher intake of fruits, legumes, vegetables, nuts, whole grains, unsaturated vegetable oils, fish, and lean meat or poultry, were associated with a decreased risk of all-cause mortality [42]. The observed patterns of healthy diets were also relatively low in red and processed meat, high-fat dairy, and refined carbohydrates or sweets. It is possible that consuming a nutrient-dense diet was associated with a decreased risk of death from all causes [42]. It seems that a high intake of fruits, vegetables, nuts, whole grains, and olive oil, which are rich sources of antioxidant nutrients, may reduce the risk of CVDs [2,4,19,20]. It is also possible that numerous individual functions of the nutrients, as well as their combined synergistic and additive effects, are crucial to understanding their protective effects on CVDs. A large majority of the phytochemicals present in the diets are redox-active molecules and therefore defined as antioxidants. It is possible that antioxidants originating from foods may work as antioxidants and anti-inflammatory agents in the body tissues, causing beneficial effects on CVDs through other mechanisms, including acting as inducers of mechanisms related to antioxidant defense, DNA repair, and cell maintenance [19,20,21,22,23,24,25,26,27,28,29,43].

In view of the complex but protective role of diet in CVDs and T2DM, it is challenging to point out a nutrient as a cause of the disease because a typical diet provides more than 25,000 bioactive food constituents [23,24]. It seems that the presence of multiple nutrients may modify a multitude of mechanisms concerned with these diseases. A recent study has reported that a high-polyphenol Mediterranean diet in conjunction with moderate physical activity was associated with lower brain atrophy, indicating that this diet can prevent neurodegenerative diseases [44]. The Indo-Mediterranean diet appears to be more suitable for Sustainable Food Systems for Food Security and Nutrition of the FAO and UNO [45]. A modified Indo-Mediterranean diet may also be advised to adolescents who consume unhealthy foods in their meals [46]. Other studies from Japan also support that an antioxidant diet rich in fruits, vegetables, whole grains, and fish, with a low fat and carbohydrate content, may be responsible for a lower risk of all-cause morbidity and mortality [47,48,49], whereas a diet rich in ultraprocessed foods and deficient in nutrients can predispose all-cause mortality [50]. Apart from flavonoids, Indo-Mediterranean diet is also rich in w-3 fatty acids with low w-6/w-3 ratio of the diets which is more effective in reducing risk of CVDs and T2DM [51]. Cocoa containing foods are also considered functional foods and nutraceuticals [52,53].

## 5. Limitations

There are variations regarding the variety of flavonoids included in studies from various countries to establish the association of flavonoid intake with risk of CVDs. There are only two randomized trials with the Indo-Mediterranean diet for prevention of CVDs [9,10] in contrast to other protective diets, which have many studies to support their cardioprotective effects [1,2,4,5,6,11,12]. The standard diet in India provides hardly 600–700 mg/day of flavonoids, which is inadequate for the management of diseases. Moreover, Indian populations typically take tea with milk, which reduces the bioactivity of flavonoids. Another limitation is that the high content of flavonoids in the Indo-Mediterranean diet is because this diet was constructed for patients, but less commonly consumed because of the transition of the populations causing reduced intake of traditional foods.

In brief, there is substantial evidence that increased intake of a Mediterranean type of diet can cause a significant reduction in CVDs and diabetes. It is also clear that the DASH diet has many similarities with the Mediterranean type of diet, without any emphasis on olive oil consumption, which can also decrease CVDs and diabetes. However, the Japanese diet may be superior to Mediterranean and DASH diets due to the greater content of antioxidant flavonoids, which is known to provide a lower risk of CVDs and diabetes, as well as longevity. Finally, the high content of flavonoids in the Indo-Mediterranean diet, similar to the traditional Japanese diet (1800 vs. 1500 mg/day, respectively), indicates that this diet can play a potential role in the management of CVDs and T2DM. The Indo-Mediterranean diet could be superior to DASH, Mediterranean, and Japanese diets due to greater food diversity and lower glycemic index and no unhealthy animal foods that are present in other diets. The high quality of the Indo-Mediterranean diet may be because of the increased content of whole grains, millets, porridge, and beans, as well as spices, which are not commonly found in other types of protective diets. The ten characteristics of a high-quality diet that are present in the Indo-Mediterranean diet appear to be basic for a better future, healthiness, and healthy life expectancy and longevity.

## Figures and Tables

**Table 1:** Comparison of Indo-Mediterranean diet with other scientific diets.

Foods	Indo-Mediterranean Diet	Mediterranean Diet	DASH Diet	Japanese Diet
Vegetables, fruits	400 g/day	High	High	High
Nuts	50–100 g/day	High	Moderate	Low
Whole grains, beans	400 g/day, high; beans millets, porridge, grams, soybean, green beans	Moderate, legumes	Moderate, legumes	High rice, soya bean, tofu
Vegetable oil	30–80 g/day, mustard oil or blend of olive oil	Olive oil, high (100 g/day)	Low saturated fat foods, oils	Low rice bran oil
Fish	100–150 g, twice/week	Moderate	Moderate	High, raw
Dairy products	Buttermilk and curd	Low fat	Low fat	Low
Wine	Not advised but allowed	Moderate	Not advised	Sake, rice wine
Spices (coriander, cumin, turmeric, cloves, cardamom)	High (50–150 g/day), coriander, cumin, turmeric, fenugreek	Not advised	Not advised	Not advised
Poultry	Not advised	Moderate	Low	Low
Red meat	Not advised	Low	Low	Low
Preserved meat	Not advised	Low	Not advised	Low
Sweets and sugar	Not advised	Low	Low	Low
Nutrients	High flavonoids, fiber, K, Mg, Ca, iron, proteins	No specific advice for protein	High K, Mg, Ca, fiber, protein	High *n*-3, K, Mg, Ca, high protein
Food diversity	Marked	Moderate	Moderate	Moderate
Glycemic index	Very low	Lower	Lower	Very low

K = potassium, Mg = magnesium, Ca = calcium, *n*-3 = long-chain omega-3 fatty acids.

**Table 2:** Ten qualities of high-quality foods.

Qualities of Foods	Examples of Foods
1. Low glycemic index	Nuts, vegetables, whole grains
2. High nutrient density.	Whole grains, beans, vegetables
3. Food diversity.	Nuts, vegetables, whole grains
4. No trans fat	Grilled foods, boiled foods
5. No/low sugar refined	Guava, apples, papaya, oranges
6. Low salt	Fruits, vegetables, nuts
7. Moderate healthy fat	Olive oil, mustard oil, nuts.
8. High fiber	Vegetables, whole grains, fruits
9. Beneficial effects on gut microbiota.	Vegetables, whole grains, fruits
10. No peroxidation of foods	Fresh foods, without frying.
Foods requiring mastication	Whole grains, nuts, fruits, fish

Adapted from Singh et al., Reference [8].

## Data Availability

Not applicable.
